# Performance Analysis of Public Safety Cognitive Radio MANET for Diversified Traffic †

**DOI:** 10.3390/s22051927

**Published:** 2022-03-01

**Authors:** Piotr Gajewski, Jerzy Łopatka, Piotr Łubkowski

**Affiliations:** Institute of Communications Systems, Faculty of Electronics, Military University of Technology, gen. Sylwester Kaliski Str. No. 2, 00-908 Warsaw, Poland; jerzy.lopatka@wat.edu.pl (J.Ł.); piotr.lubkowski@wat.edu.pl (P.Ł.)

**Keywords:** MANET, dynamic spectrum management, cognitive radio, services, telecommunication traffic, computer modeling, simulations

## Abstract

This paper presents properties of a mobile ad hoc network (MANET) with dynamic spectrum management (DSM) and is devoted to the concept and implementation of the new traffic engine that is used in a High-Fidelity simulator of MANET with cognitive nodes for special applications. The communication traffic generated by each node is defined according to its role in the hierarchical structure of the operational scenario, determining its priorities, permission to use particular real time and non-real time services. The service usage is a source based model, defined in the user’s profile containing its statistical properties, describing periodicity, duration and randomness of traffic generation. The overall traffic generated by the node is a combination of traffics related to specific services. Their statistical parameters are based on real exercises results. The model was defined in the Matlab environment and next verified using the MAENA simulator for complex, operational scenarios. The achieved results show that use of both central and distributed DSM provides a better performance of the MANET network with complex traffic.

## 1. Introduction

In the last two decades, there has been a continuous increase in demand for telecommunications services provided to mobile users (people and devices). Links between moving devices dynamically change the network configuration. This kind of networking is widely used in many areas like the Internet of Things (IoT), Intelligent Traffic Systems, Vehicle-to-Vehicle linking, especially in public safety systems including first responder and military applications and is referred as to Mobile ad hoc Network (MANET). This technology enables connecting mobile devices without the use of access network infrastructure, and connections between nodes are made directly or through other nodes, so MANET is a wireless, multi-hop, self-configuring network with mobile nodes.

A fully implemented MANET can be a powerful way to enable connectivity in networks of very high complexity and for a large variety of operations supporting public safety (e.g., rescue, police, military) [1]. 

The diversity of tactical operations, variable speed of the nodes, and the wide range of platforms used presents the challenges to the full and seamless deployment of tactical networks. Brubank et al. in [2] identified the key challenges for military tactical networking, pointing to the capabilities of the MANET technology. They claimed that the use of an ideal MANET in a tactical space requires research in the field of solutions including medium access control (MAC), management, security, and routing. Many of these questions are characterized in [3]. Here, several kinds of MANET are pointed out: Vehicular Ad Hoc Network (VANET), Inter-Vehicles Communication (IVC), Vehicle to Roadside Communication (VRC), and Inter-Roadside Communication (IRC), Internet-based MANET (iMANET) where mobile nodes are self-configured, and Intelligent VANET (inVANET), that can also solve a problem of the available spectrum resources scarcity.

Mobile Ad-hoc Networks with Dynamic Spectrum Management (DSM) including Artificial Intelligence (AI) applied in Cognitive Radio (CR) nodes are the most promising concept technology supporting complex requirements to provide communication services in a heterogeneous C4ISR (Command, Control, Communications, Computers, Intelligence, Surveillance and Reconnaissance) system environment. According to Haykin’s definition of CR [4], the Dynamic Spectrum Access (DSA) is recently studied in [5,6,7]. MANET with Cognitive Radio nodes (CR MANET) is recently investigated for Opportunistic Spectrum Access (OSA) applying Artificial Intelligence and Machine Learning (ML) algorithms [8].

The efficient use of this technology requires special computer tools ensuring communication system planning, dynamic management, and evaluation of the quality of communication to support the military operations. Such a tool should include procedures enabling networks modeling and simulation to assess network technical and operational performance [1]. This assessment is as accurate as used models are reflecting the real conditions on network operation, including expected user traffic.

To investigate the behavior of the whole network, especially used to support public safety, its model should consider network architecture, including the structure of information flow between users, connections between senders and recipients, the structure of users organization, types of messages sent, and the way of their transmission. 

High fidelity simulator for tactical networks based on Cognitive Radio was elaborated in CORASMA (Cognitive radio for dynamic spectrum management) multinational program managed by European Defense Agency (EDA) and sponsored by seven countries: Belgium, France, Germany, Italy, Poland, Portugal, and Sweden. The main objective of this simulator was validation of various cognitive solutions at a 5th technology readiness level (TRL) [9]. In [10], the specific algorithm for distributed channel selection for hierarchical MANET was presented. Based on the experience of CORASMA, a laboratory and real demonstrators have been built to test DSM procedures [11]. The performance monitoring of CR MANET with DSM was characterized using the proposed set of evaluation metrics in [12]. A solution containing our waveform used in CR nodes is presented, and results obtained in a real environment are discussed in [13]. 

This paper presents the results related to MANET properties obtained from the new HiFi MAENA Simulator for CR MANET built in a frame of just completed multinational project MAENA (Multi bAnd Efficient Networks for Ad hoc communications) also managed by EDA. The CORASMA simulator and results were used as background information for MAENA, in which the multi-band CR MANET with dynamic spectrum access DSA models have been developed for the near-real military tactical environment with heterogeneous traffic. 

The article is organized as follows. In Section 2, the issue of CR MANET for public safety is characterized, and the architecture of the network model for hierarchical organization of command and control is presented, with the description of two-stage spectrum access including central and local DSM. Because one of the main research problems is proper traffic generation, the users’ profiles and communications services are characterized in Section 3, and the source traffic generator is described in Section 4. Next, the simulation environment and scenario are presented in Section 5. Section 6 contains presentation and discussion of performance characteristics as results of conducted simulations. Finally, we conclude our works in Section 7.

## 2. Cognitive Radio MANET for Military Tactical Networking

### 2.1. Military Network Architecture

According to Brubank [2], MANET is a key element of the Global Information Grid (GIG) that provides information superiority on the battlefield, because military MANET consists of a set of various participants interacting to complete the mission. In this area, the Network-Centric Warfare (NCW) is a paradigm to provide ubiquitous network access for any time and anywhere continuous communications. 

**Figure 1:**
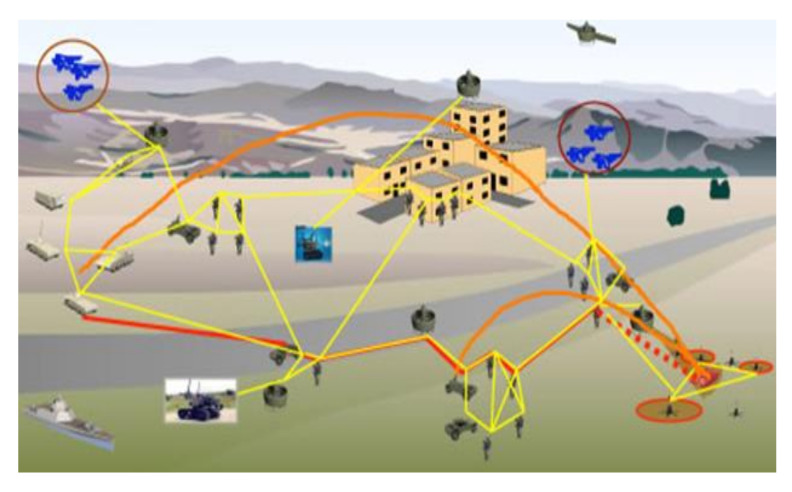
An example of the battlefield environment.

Figure 1 shows schematic connections between various participants in an example battlefield. Generally, the operation is highly dynamic and consists of a variety of network elements, various terrain conditions, and unpredictable adversary actions, including jamming and improvised explosive device (IED) use. The main constraints of the tactical military environment are [2]: extreme mobility, intermittent connectivity due to mobility, risk of information capture or its compromise, operational security, poor channel quality, limited bandwidth, intermittent connectivity from terrain (environment), platform constraints (size, weight, power), environmental requirements limiting capability.

**Figure 2:**
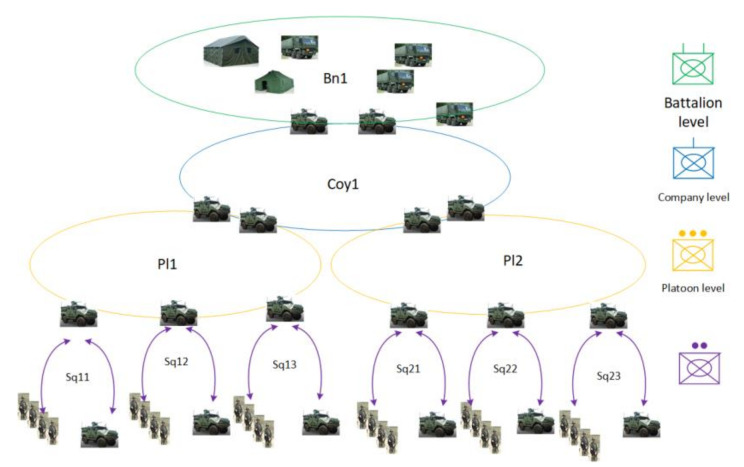
Hierarchical combat organization.

To apply the MANET in a tactical area, it is necessary to consider the type of forces deployed and their organization that is described in Order of Battle (ORBAT). Usually, command and control are organized hierarchically as shown in Figure 2. Here, for battalion operation, a four-level command structure is used including battalion, companies, platoons, and squads. Different networks ensure communication between nodes at a specific level, and between different levels of command, ensuring a two-way flow of information in the system, taking also into account the diversity of data sources and cooperation between Allied units. Specific networks are created for command, combat management, reconnaissance, logistics, medical support, and cooperation between various forces. Diversification of types of transmitted information, used frequency bands, different waveforms, prioritization of nodes and services creates a heterogeneous environment with the high complexity of telecommunications traffic. To avoid the discontinuity of communication caused by terrain obstacles or long range, supporting nodes placed on the UAV (drones) can be also used. 

On the battlefield, wireless sensors are increasingly used, starting from recognition systems and environmental monitoring to personal soldier health monitoring systems. Wireless sensors perform more and more complex functions, typically creating wireless sensor networks (WSN). To deliver the NCW capabilities to the forces, the MANET must fulfill four general requirements: strong connectivity, very high throughput, effective security, and survivability [14].

Although the issue of security goes beyond our solutions in the MAENA program, it is particularly important in terms of the threat of cyber attacks, as indicated in [15]. It covers many aspects, from building trust of information sources, data confidentiality, resistance to various cyber attacks and jamming. These questions are recently very intensively studied and published. Some of them analyze the robustness of routing protocols for MANET, proposing modifications of the classical algorithms such as OLSR [16], or new hybrid solutions [17], Key Management Method [18], and method using efficient trust establishment [19]. Comparative analysis of chosen routing algorithms security is presented in [20,21,22], and the intrusive detection methods using ML algorithms are proposed for MANET by [23].

### 2.2. CR MANET Modeling and Simulation

In general, a model is a simplified representation of the system used in the investigation, reflecting major characteristics of the real system. The complexity of the model depends on the mapping details of the real system into the computing environment according to the investigated problems. High-level modeling using the graph theory is described in [24]. Here, Erciyes et al. discussed several graph models of MANET to analyze various aspects of MANET like topology control, interference analysis, and so forth. As such models have many constraints, the authors of [24] present the comparative analysis of different simulation tools and mobility models for MANET. 

For building simulation models, experience from existing solutions in commercial tools like OMNET++, INET/MANET, OPNET, and so forth, is often used, considering the specific properties of MANET-based systems. Each of these tools enables the modeling of specific aspects of MANET and these limitations are discussed in [25,26]. To enable modeling of the full stack of MANET and take into account the influence of the radio environment the construction of a dedicated simulator is necessary. 

The CORASMA simulator had been used to investigate various aspects concerning DSA in CR MANET at the tactical level. This simulator was built using OMNET++ as a background to develop a network consisted of nodes equipped with Cognitive Radio functionality. To model the behavior of a real MANET system, many procedures have been developed. Some routines are taken from or adapted from open source INETMANET. In CORASMA simulator, the CR MANET functions involved spectrum sensing, spectrum management, and radio resources optimization that were elaborated and studied in real operational scenarios. Detailed modeling of nodes behavior using specific waveform, propagation conditions, environment impact including interference from Non-Cooperative Nodes (NCN) like TETRA transmitters and jammers, creates a HiFi simulator to model and compare various aspects of CR designed for use in tactical battlefield systems. The main constraint is to use one Wide Band (WB) waveform to High Data Rate (HDR) transmission in Ultra High Frequency (UHF) spectrum band.

**Figure 3:**
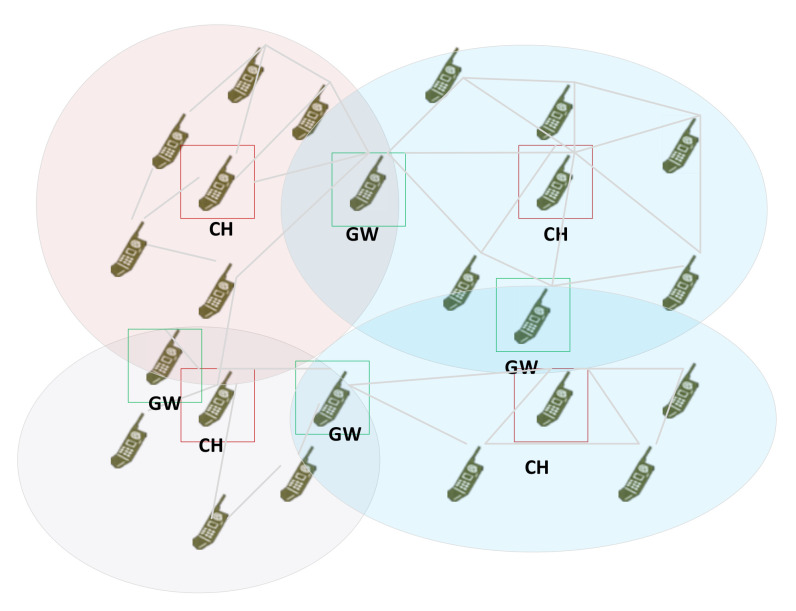
Cluster architecture.

In CORASMA, the network is modeled as a set of dynamically changing cluster structures (Figure 3), in which three kinds of node’s functionalities are defined. Cluster Head (CH) is the main node automatically chosen within a group of neighboring nodes, that managed clusters’ Ordinary Nodes (ON), some of them belong to two or more clusters and they perform the gateway function between clusters.

In CORASMA, two clustering methods were deployed: static and dynamic. In the first one, the clusters are constructed based on command hierarchical model with commander radio as a cluster head. Apart from that, in [27], we proposed a distributed clustering method based on the multi-criteria decision that uses the fitness function, which combines weighted values of the main node and network parameters. 

### 2.3. MAENA Simulator Overview

A hierarchical model with several levels of command and control is usually utilized in such applications. Such architecture can consist of both people and machines, and transmission nodes can be located in various mobile platforms (handheld, backpack, light cars, heavy vehicles, drones, UAV, helicopters, planes). It was a motivation to extend CORASMA simulator capabilities in MAENA project, to support multi-band operations, cluster-based and flat networks, coordinated and distributed DSM. Each radio node can be used by a single user or group of users that are characterized by their traffic profiles. The set of users is organized in a multilevel, hierarchical structure as shown in Figure 2.

**Figure 4:**
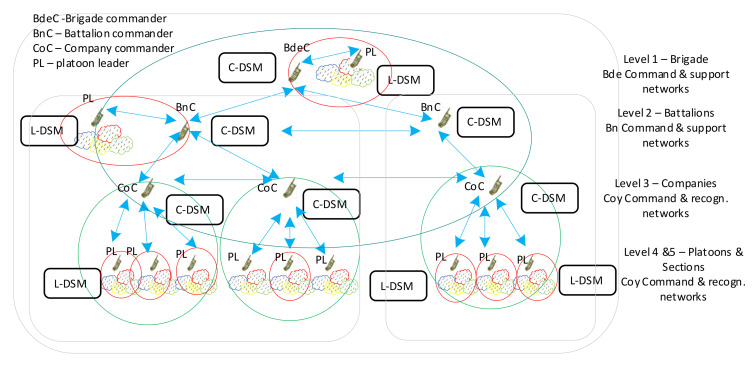
General architecture of DSM in MAENA.

The main feature of the MAENA simulator is the ability to model and test various solutions, implemented within the next generation radio concept, including waveforms, protocol stacks, applications, and variable policies regarding their behavior and way of use. They also include solutions devoted to network management including both local DSM and coordinated, central DSM [8]. Figure 4 shows the general architecture of MAENA. As it was shown earlier, this network in general consists of two types of nodes: Cluster Heads, and Ordinary Nodes. 

Each ON contains spectrum sensing module, enabling sequential monitoring of a predefined set of frequencies. To detect presence of the signal, the sensing is performed in silence periods of the transmitter [13]. Detector parameters are flexible and are controlled by sensing policy, defined by the network manager. 

Architecture of the CH is more complicated. From one side, it acts as local DSM entity, but from the other it is also connected to central DSM. Local DSM is responsible for controlling the spectrum sensing process, collecting the sensing results from ONs, data fusion using evidence theory, channels utility evaluation using Q—learning [28] and greedy algorithm for radio resources assignments for own cluster. 

The central DSM [8] is a hierarchical structure of entities providing coordinated assignment of spectral resources to all networks and clusters, assuming minimum level of mutual interferences and based on positions of all nodes. Before mission, it provides initial assignments, and during the mission the assignments may be updated because of nodes mobility, interferences and intentional jamming.

To set up and conduct experiments using a simulation-based approach, all these elements must be combined and harmonized by a scenario definition tool that enables description and modification of the simulation scenarios including the introduction of global parameters such as the number of networks, propagation, and networks parameters. However, the most important is the definition of order of battle that is, the military structure which must be simulated. The OoB includes setting communications parameters like spectral masks, air interfaces, waveforms, DSM structure, sensing, and other solutions. Initial routes are also created using the digital map of terrain (DTM) also including the digital elevation model (DTEM) and terrain obstacles like for example, buildings, forests (and other obstacles clutters defined by the user). User traffic flows to be forwarded across the network are defined as well. Finally, the nodes’ initial positions and mobility are set up. An additional feature available in the simulator is the ability to create non-cooperating nodes, which generate interference degrading the communication.

The simulator uses the Human Machine Interface (HMI) to define the scenario, military units’ structure, communication networks, and their parameters, as well as to load a map background and the layout of defined nodes on the loaded map. Currently, the simulator allows defining a node equipped with up to four air interfaces supporting communication in UHF or VHF channels. These nodes are associated with vehicles, soldiers, UAV’s, UGS’s or UGV’s. Two basic waveforms are used for UHF and VHF connections, however, their parameters can be modified, or a new waveform can be created. Basic waveforms are further extended by integrated solutions with specific functions as clustering, resource management, DSM algorithms, and sensing solutions. 

Each node generates a signal of the used waveform as a string of IQ samples that are converted into received samples according to the assumed propagation model. The specific models are used for ground-to-ground, ground-to-air, air-to-air links including also co-site and co-vehicles effects, small scale fading, and so forth. So, the shadowing and multipath phenomena can be switched on or off in our model. To validate some of them a measurement campaign was conducted in suburban environment [29]. 

The simulator enables the generation of nodes’ movement by defining their routes on the map. The first set of initial positions enables communication within each communication network. Additionally, the user may explicitly define routes to allow communications between nodes in different communication networks.

For such a prepared scenario, it is possible to start the simulation with the dedicated simulation seed. The obtained results are presented in a graphical form to facilitate their analysis. The elaborated tool enables carrying out further research concerning the defined structure of military units implementing the assumed operational scenario. 

In the following section, we present the results of the MAENA simulator obtained for the useful military scenario of peacekeeping operation provided by military troops inside the city in the situation of active opponent actions like fire attack, using jamming, and IED.

## 3. Source Traffic Characterization

### 3.1. Traffic Models

Proper traffic modeling is a basic requirement for accurate network assessment and recent works concerning traffic modeling are usually focused on selected aspects of the network. Some papers discuss mathematical models for theoretical traffic description [30,31]. Many studies are about matching theoretical models to one class of services like Voice over IP (VoIP) [32], VoIP and Video over IP (VioIP) over LTE Networks [33], or Push-to-Talk (PTT) voice transmission [34,35]. Because of the high unpredictability of the mobile environment, for traffic modeling, probabilistic models are extensively used. The Pareto distribution is proposed in [36,37] for modeling complex traffic that consists of voice, video, data, and telemetry messaging. As shown in [3,38], in MANET, the routing protocol also has a significant impact on traffic in multi-hop transmission.

Traffic modeling in military MANET should consider both the characteristics of services taken from the exercises experience and the properties of technical solutions in the network. Such a model should consider service-specific traffic parameters, such as required bit rate, the average duration of conversation and transmitted video, average intervals between calls, number of generated events of a given service type, authorization to service access, distribution of pseudo-random user activity, priorities for individual events generation, the randomness of connections (addresses of senders and recipients), queuing procedures, and so forth. Some data are sent frequently, like for example location and identification data usually called Blue Force Tracking (BFT). Most of them have a probabilistic nature.

### 3.2. Services in Safety Support MANET

The military MANET main services characteristics are shown in Table 1. In general, traffic can be divided into two classes: real-time (RT) and non-real-time (NRT) depending on the land cover permissible time delay between sender and recipient, some of them are connectionless (CL) or connection-oriented (CO). Three kinds of protocols are used in the network: UDP—User Datagram Protocol, RTP—Real-time Transport Protocol, TCP—Transmission Control Protocol.

The next subsections contain a more detailed description of these services with a short discussion of theirs traffic models.

### 3.3. Voice Transmission

The low data rate of 2.4 kbps using MELP (Mixed Excitation Linear Prediction) coding is mainly used in VoIP transmission in special radios, but the high data rate 16 kbps CVSD (Continuously Variable Slope Delta) modulation is used in legacy radios for Push-to-Talk (PTT) conversation.

PTT is a multicast semi-duplex service like a walkie-talkie (one is talking, the others are listening). This is the primary way of making voice calls on the battlefield, proposed also for ad hoc cellular networks in [34,35] as mission-critical services.

The packet streams created by the voice codec are transmitted between sender and recipient in unicast mode or a group of recipients in multicast mode. According to the real operational situation, it can be assumed that two kinds of streams are used in VoIP namely: short voice command message up to 10 s (VoiceA), and long voice reporting message more than 10 s long (VoiceB).

### 3.4. Video Transmission

Video over IP systems use existing video codecs to convert a video material into a bitstream and then send them in the form of a stream of IP packets, which is usually transmitted using a certain variant of the RTP protocol. The image size depends on the resolution of the camera. According to the operational requirements, there are three major types of cameras:

- Personal camera with 1 Megapixel resolution.
- A camera on unmanned aerial vehicles and unmanned ground vehicles with the resolution of 2 Megapixels.
- A device that is equipment of ground vehicles with 4 Mpixel resolution.

Assuming that the compression standard used is JPEG 2000, three ranges of data rates have been obtained for each node type as shown in Table 1.

### 3.5. Blue Force Tracking

Blue force tracking (BFT) is a system that mainly supports two processes: Situational Awareness (SA) and Command and Control (C2) between deployed troops. Messages regarding SA are broadcast, while C2 traffic is mainly based on point-to-point transmission. The restrictions are influenced by several variables, such as uplink and downlink data rates, the number of users of the network, the amount of data exchanged, processing techniques, the amount of information about situational awareness and transmission policy.

Nevertheless, it can be assumed that this service supports two types of messages. First of all, there is BFT user message which is sent by each BFT node to the BFT server. Secondly, there is BFT server message, which is sent by the server to the BFT nodes. The BFT user message size is about 2 kB. The BFT server message size is calculated by multiplying 2 kB by the number of supported BFT objects (nodes).

### 3.6. Alerts

Alerts are very short multicast messages sent by a particular node occasionally according to temporary operational situations, for example, massive artillery fire, life-threatening, and so forth.

### 3.7. Chat and Email

The chat and the e-mail traffic profiles are characterized by a message size and an attachment size (usually in email). Chat is the most popular transmission used to NRT conversation between users at the digital operational area. As real military exercises have shown, it can be assumed that three message values are used (Table 1): short messages (SMS), medium free text, and formatted messages up to 10 kB long.

E-mails can be characterized as short or long NRT messages dependent on the attachment: 5–50 kB without attachment, and up to 2 MB with long attachment.

### 3.8. C2 Messages

The main purpose of C2 is to provide commanders a possibility of data exchange, usually used in an automated Battlefield Management System (BMS). These data can be various, including maps, tactical situations, pictures, data from sensors, and so forth. A very short characterization of BMS messages is shown in Table 1.

BMS messages are used to submit situational awareness to higher levels. They collect information about the location of members at particular levels and Local Operational Pictures (LOP) that are to be delivered to superiors. During the collection phase, the message is the sum of individual reports. The Common Operational Picture (COP) messages are used to pass information from higher levels of the command chain to subordinates. In general, it can be assumed that COP at the sender level is enriched by about 70–100% of data provided by neighboring units.

## 4. Traffic Modeling

In general, all works concerning traffic modeling and analysis can be categorized according to three criteria [39]:

1. the goal of the analysis;
2. the point where the network traffic is captured (henceforth simply referred to as a point of capturing);
3. the targeted mobile platforms.

Traffic modeling is a very complex issue. In [39], Conti et al. identified thirteen goals of analysis that are sorted into three areas: traffic characterization, application identification, and usage study.

### 4.1. Assumptions

It is assumed that each node generates the traffic that consists of several services according to scenario requirements such as for example, kind of network (command, recognition, support, cooperation, data, …), the node’s role in the network (commander, subsidiary, neighbor), phase of operation, and so forth. The Constant Bit Rate (CBR) traffic can be used, but most traffic is generated as Variable Bit Rate (VBR) traffic, that uses UDP or TCP protocols.

The traffic generated by a particular node can change according to its role in the scenario and policy, which requires a definition of a large set of used traffic profiles. The concept of traffic profiles is also proposed in White Paper “A 5G Traffic Model for Industrial Use Cases” [40].

In our solution traffic profiles are used to set up general parameters of traffic generator according to the following service’s attributes:

- Type of transmission—streams or blocks
- Type of connection—unicast, multicast
- Destinations addresses (NodeID, IPAddress, …)
- Type of service—real-time (RT) or NonReal Time (NRT).
- Emission timing—transmission duration (mean, variance), periodic (period mean value, period variance), duration between transmissions, Start/End
- Message size (mean, variance)
- Required bit rate (mean, variance)
- Protocols (TCP, UDP, RTP)
- Service priority

In general, most of these parameters can be defined as random values with assumed distribution, and each part of the traffic is generated as a discrete-time event. The traffic generated by the node is a sequence of events ordered in a queue, according to the rules including MAC procedures.

### 4.2. Traffic Modeling

Definition of traffic model is a complex process of building and verifying the mathematical description of the traffic [41]. This process includes the following steps: observing, modeling, deducting, measurement and verification.

As it is stated in [30], traffic analysis is a vital component to understanding the requirements and capabilities of networks. Traffic models are used in two fundamental ways: as part of an analytical model or as discrete event simulation.

Some traffic patterns are identified in [40] for industrial use cases in 5G. They are deterministic traffic (aperiodic and periodic including time-triggered periodic) and stochastic non-deterministic and burst traffic (periodic and aperiodic). Mixed source traffic has been adopted for the modeling of scalable multi-service networks with many features as priority access, QoS demands, delay-tolerant and non-tolerant devices, and so forth [42].

Traffic models fall into two categories: source and aggregated traffic ones [42]. The first one aims at modeling traffic at the output of information sources (e.g., voice, video, e-mail, chat, short data). The second category contains aggregated traffic models describing traffic in links, network, and also recipient traffic.

There is no single model that can be used effectively to model the traffic in various kinds of networks. A simple Poisson model is widely used but is limited to heavy-tailed traffic if the number of sources is infinite and the traffic arrival is random. For multiple access systems, aggregated traffic models using Poisson binomial distribution are widely deployed [30]. Such a model is proposed in [43] for traffic modeling in cellular GSM, where calls use two models: the Poisson arrival rate and exponential interarrival rate. Laner et al. in [44] proposed specific Coupled Markov Modulated Poisson Process (CMMPP) for machine to machine (M2M) communication, if the massive amount of devices have to be modeled. This source model has many advantages compared to 3GPP aggregated models proposed in [42] for such types of communications. The MMPP (Markov Modulated Poisson Process) is also proposed for VoIP traffic in which aggregating “On-Off” model is used for voice packetization, with an assumed CBR source case [32]. The four-state discrete-time model is introduced and discussed in [45] for conversational voice traffic, additionally including mutual silence and double-talking in Point-to-Point (P2P) communication.

The Poisson distribution is mostly used for heavy traffic modeling. If traffic has a burst form, the Pareto distribution is preferred, because it takes into consideration the correlation of packets arrival time. Such model is described in [37] for multimedia traffic, including voice, video, data, and telemetry data transmitted in PLC (Power Line Communications). Simulation provided in [36] shows that Pareto model is well matched to the burst Internet traffic with an exponential probability distribution.

For more complex sources of traffic, the global data stream may exhibit high-order statistical proprieties that are very difficult to capture, so source traffic modeling is preferable to use [44]. Doci et al. in [46] classified traffic as real and synthetic traffic models. In [41], Haryadi describes the method how to create the mathematical model of traffic distribution, using an implementation of Birth and Death Process. To obtain the best fit of traffic model, selected from the library of candidates, the method of real-time measurement-based, for traffic model classification, is proposed in [47] and the design parameters for the best classification are discussed.

The source traffic generator concept is assumed in the proposed solution, as described below.

### 4.3. Source Traffic Generator

**Figure 5:**
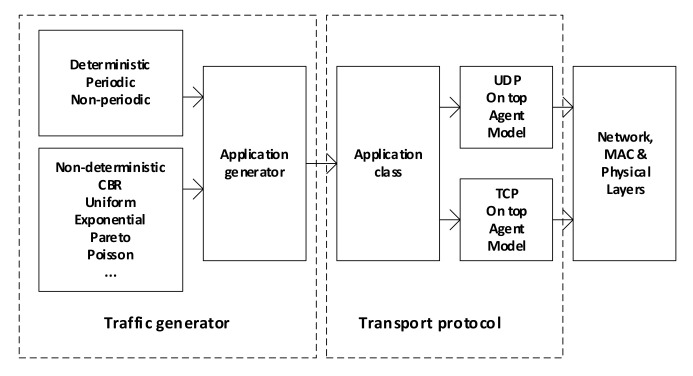
Node model with traffic generator.

When the simulation tools are used (e.g., OMNET++, NS-2), the source traffic generator is viewed as two layers approach including the traffic generator and implemented transport protocols [46]. Figure 5 shows the architecture of the network’s node using such a proposal. Here, the deterministic and probabilistic models are proposed in traffic generators that used UDP or TCP protocols depending on the user’s service.

**Figure 6:**
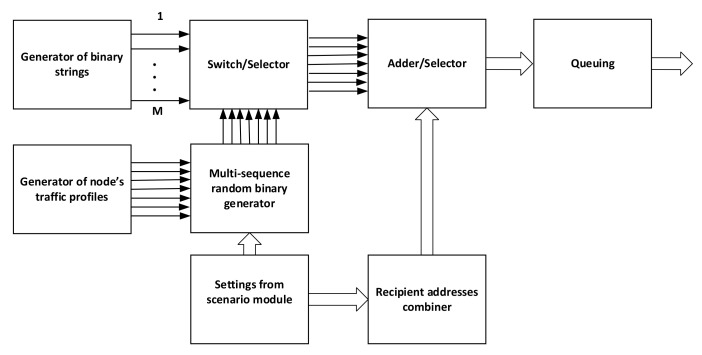
Generator block scheme.

In our concept, the traffic generator is composed of several specific modules as shown on Figure 6.

In the proposed approach, each module needs some input data and simulation settings/seeds. Most of them are coming from the scenario definition module that splits scenarios into operation phases, including network composition, mobility, events, nodes’ services accessibility, and so forth. The traffic profile module is used to set up general parameters of the generator, according to the service profile and each profile may consist of several services according to the scenario requirements. Separately, binary strings with predefined parameters are used as services data, switched ON/OFF by a multisequence random binary generator. They are combined/multiplexed with recipient addresses generated by the selector.

**Figure 7:**
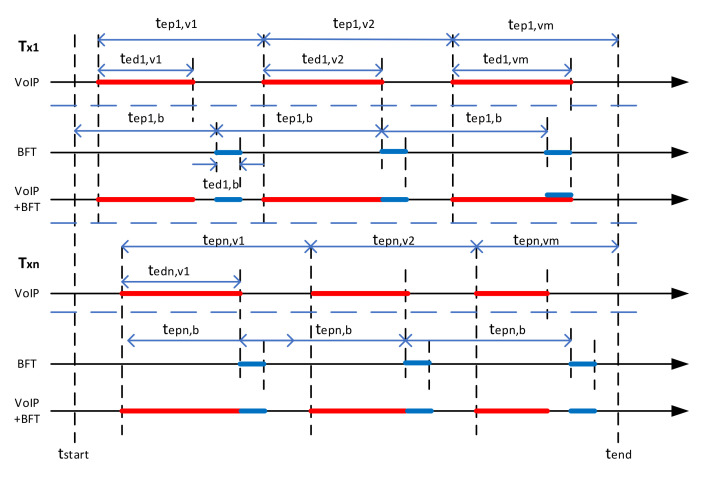
Traffic parametrization.

The implementation in MATLAB is described below. The traffic parametrization is characterized in Figure 7 as an example for two transmitting nodes in the network using two types of traffic: VoIP, and BFT.

Each elementary traffic is described by: emission period (tep), emission duration (ted), the quantity of data to be transmitted (data rate r, message size m). For TCP services the message size is used, otherwise, UDP services can be described both by message size and data rate. In the presented example, emission period and emission duration are constant for BFT, but for VoIP, they are randomly changing with statistical distribution described by general statistics: probability distribution function (PDF), mean value (M), and variance (V). The simulation time is determined by the time of start and the time of the end.

### 4.4. MATLAB Implementation

Taking into account that network users create a hierarchical structure, it can be assumed that traffic generated by a node depends on its category (level of command). In our scenario, five users categories are assumed (see Figure 2): 1st level (battalion) commander—level (company) commander—3rd level (platoon) commander, 4th level (section) commander, 5th level unit (soldier, recognition RECON UAV, communication COMM UAV, UGS (Unmanned Ground Sensor), and so forth. Each node traffic is a combination of components corresponding to various services, using the model of traffic classification based on scenario events.

For each category of nodes, the traffic profile is defined by the selection of available services. It can be set manually or the defaults can be retrieved from memory. Priorities of node and service can be set up if needed. The services parameters for the particular category of nodes can be displayed by using the window for setting the parameters of the services for the selected node category. The values of these parameters can be determined as data set estimated from lessons learned [1].

**Figure 8:**
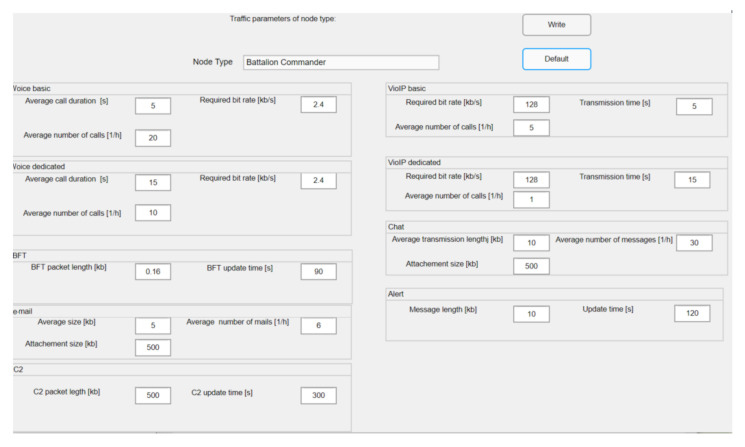
Traffic parameters definition.

Figure 8 shows an example of a profile set up for the Battalion Commander. The parameter values can be introduced as default or put manually and saved by the button “Write”.

**Figure 9:**
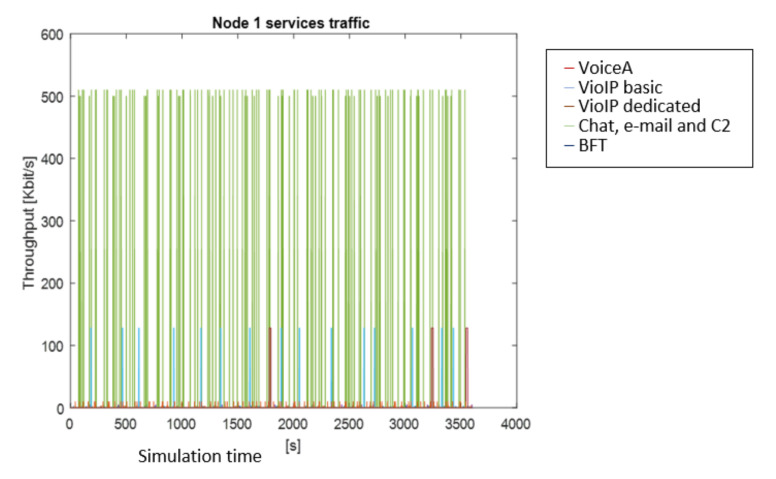
Traffic generated by particular services of Node 1.

**Figure 10:**
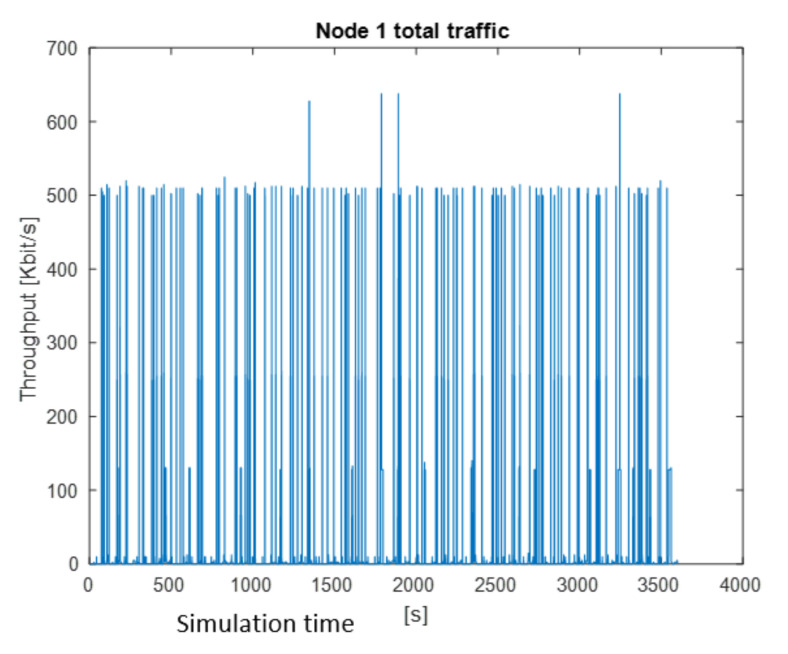
Total traffic generate by Node 1.

**Figure 11:**
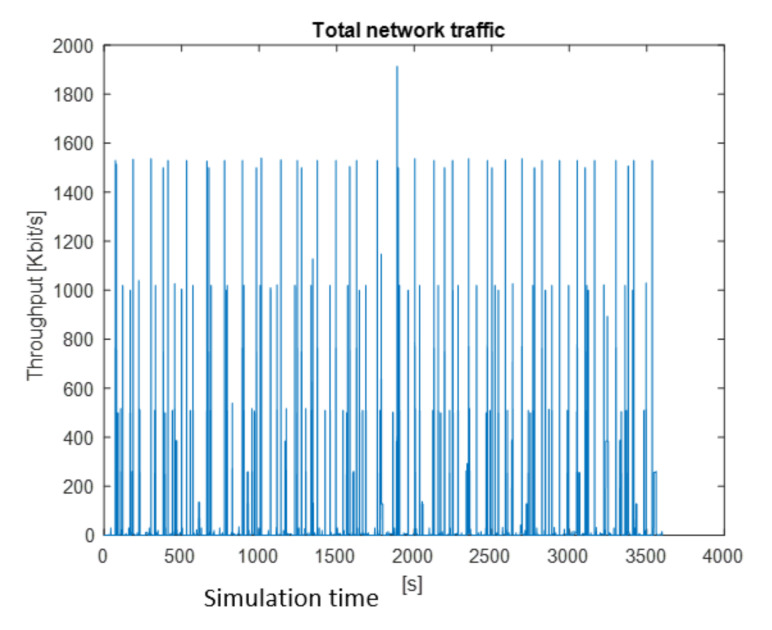
Total traffic generated by all nodes.

After the simulation, the results are shown in graphical and numerical forms. Some examples of results obtained for the presented simulation in MATLAB are shown in Figure 9, Figure 10 and Figure 11. The figures present the changes of a generated stream versus simulation time.

Figure 9 shows the traffic generated by particular services for the Node 1 profile. Different colors are dedicated to particular services. As an example, the blue line shows the Video basic traffic, brown—Video dedicated messages, whereas the red one—VoiceA traffic. The green line presents chat, C2 and e-mail messages, the navy blue one is for BFT. The maximum value is 512 kbps, the frequent red line shows a 2.4 kbps value for the voice traffic.

Total traffic generated by Node 1 is shown in Figure 10. As we can see, the value of 500 kbps is exceeded very frequently. The maximum value is over 600 kbps, minimum 2.4 kbps.

Traffic generated by all nodes is shown in Figure 11. The maximum value is 1792 kbps. Three characteristic values are also observed: 1536 kbps, 1024 kbps, and 512 kbps.

Presented results show the traffic generated by particular nodes having various profiles. To examine the performance of the whole network this model should contain an additional block to generate the recipient’s node’s IP addresses. Here a random number generator pooling recipient’s address from the network IP addresses space can be used.

## 5. MAENA Used Simulation Scenario

**Figure 12:**
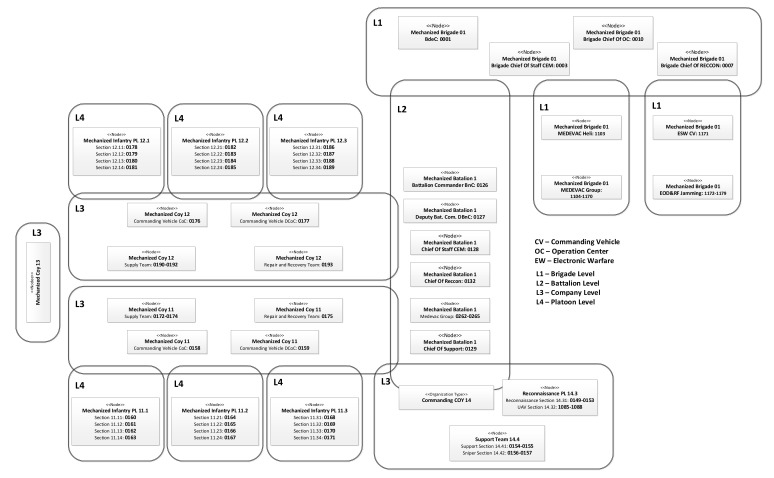
The order of battle for the operational scenario.

The simulation of the proposed operational scenario (signed as MBv5) allows verifying the performance of the cognitive MANET under conditions close to the military operations. The scenario includes one communication system including 183 communicating nodes. The Order of Battle for the MBv5 is illustrated in Figure 12.

The availability of the frequency resource is a crucial aspect of the operational scenario. For simulation, the best case was introduced in which we assumed the availability of 1500 frequencies for the VHF and 40 frequencies for the UHF. Considering that the operational scenario involves both UHF and VHF, we define 4 UHF and 28 VHF networks. The total number of Air Interfaces provided for the MBv5 is 419 (183 for the UHF band, 236 for the VHF band).

The main effort of scenario preparation was related to mapping the tactical situation onto the simulation model. It was assumed that a propagation model in $d^{-3.5}$ (with d the distance between a transmitter and a receiver) will be used in the operational zone. Coefficient 3.5 was assumed as the mean value for urban environment [29]. The goal of this approach was to simplify the network model, to eliminate influence of the complex propagation conditions on cognitive algorithms, because it enables simpler inference about the behavior of cognitive processes. As the next step, simulation with complex propagation model, considering DTM, DTEM, obstacles and buildings, land cover and multipath propagation is planned.

**Figure 13:**
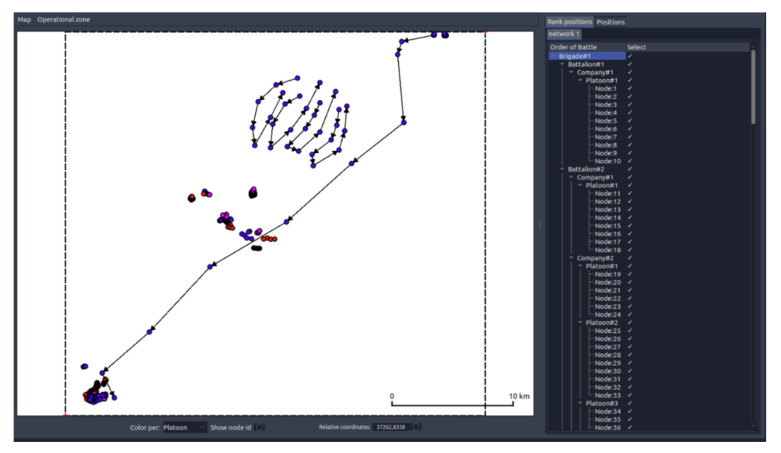
Position and trajectory for MBv5.

Concerning several nodes, routes of movement in the terrain have been planned to achieve the starting positions for operations, as well as to perform tasks resulting from the operational situation adopted for MBv5. It concerns mobile nodes such as UAV (Unmanned Aerial Vehicle) or CN (Communication Node) belonging to platoons carrying out tasks in urbanized terrain. The adopted trajectory of mobile CNs has a simplified character, which results from the need to limit the amount of information to be processed during the simulation (Figure 13).

For the performance analysis the following traffic flows have been proposed:

- PTT;
- UDP Basic;
- BFT.

**Figure 14:**
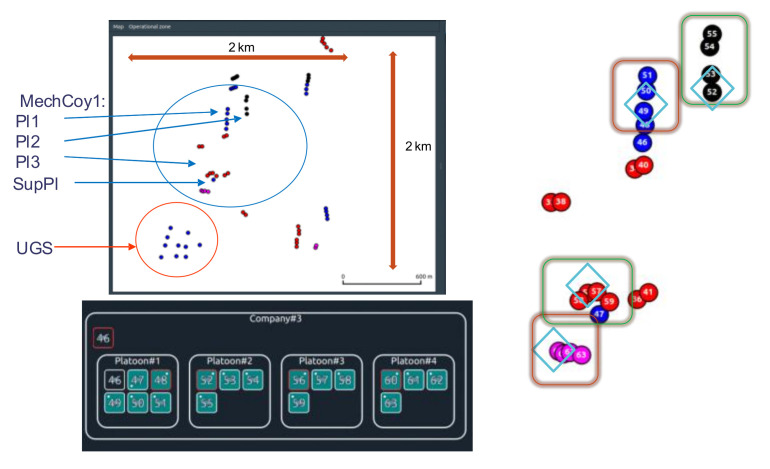
The communications network for UHF sessions (Comp_BMS) for the MBv5 best case.

In such a case, the company (Mech Coy 1) networks are further presented. Here, MechCoy operates on an area of 2 × 2 km. The communication network consists of 36 CNs, that were deployed as shown in Figure 14.

The PTT traffic is supported within VHF Communication Networks and the platoon commanders are the source of the traffic. One session took 5 s and is established every 10 s. For simulation, 19 PTT sessions were established with the assumption that one session is associated with the last VHF Air Interface.

The Communication Network is handling 36 point-to-point data traffic flows, based on the basic UDP traffic generators. The 27 UHF flows are 1-hop Intra Cluster flows and have a 40 kb/s rate. Seven flows belong to the VHF (VHF data) and each of them has a 500 b/s rate. Additionally, two VHF flows have a 160 kb/s rate.

**Figure 15:**
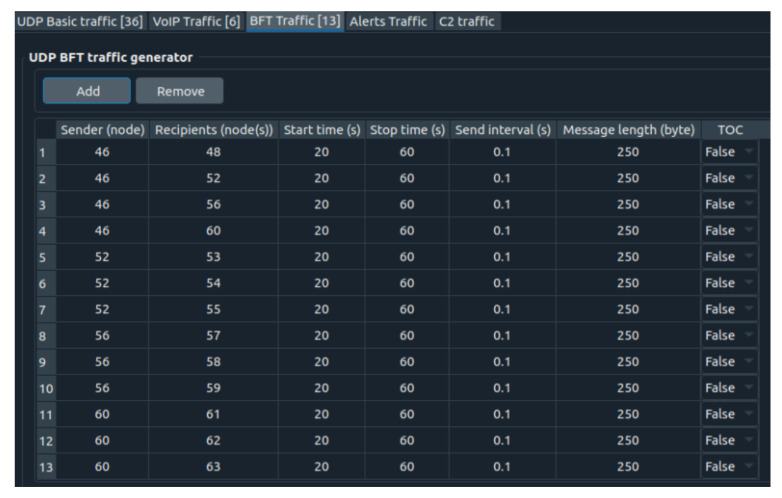
BFT traffic flows.

In addition to the PTT and UDP flows, some BFT traffic is also injected into the network. Each of them has a 20 kb/s rate (Figure 15).

A set of simulations was conducted for performance analysis of the MANET with cognitive nodes. The 100 s of MBv5 simulations were performed for three cases of traffic. In case 1, only PTT sessions were generated, while in case 2, PTT and UDP sessions were used, and finally, PTT, UDP, and BFT sessions are performed in case 3. Table 2 presents the main parameters of simulations.

## 6. Performance Analysis

### 6.1. Metrics

Two kinds of metrics have been adopted in MAENA that are defined as Metrics of Performance (MOP) and Metrics of Effectiveness (MOE). MOP set involves the measures of technical parameters used for evaluation of the performance of communications networks such as for example, Packet Error Rate (PER), transmission delay, delay jitter, throughput, and so forth. considering their probabilistic nature. While MOEs characterize the overall efficiency of the system from the user’s point of view, for example, the probability of transmission success, Quality of Experience, and so forth.

The very wide overview of metrics proposed for the assessment of CR networks is shown in [48]. Many of them were adapted for evaluation of CR MANET during CORASMA project, as it was presented by us in [12]. The metrics used in this project were implemented in MAENA, where many metrics related to the assessment of additional functionalities have been added, such as spectrum utility and effectiveness by DSM.

A very limited choice of measures is used usually to assess the performance of CR-based networks. For example, in [49] the interference time, propagation delay, end-to-end delay, and throughput were used to evaluate the CR system model based on the IEEE 802.22 standard. The traffic throughput, SINR, and achievable data rate related to the modulation technique were used for performance evaluation of QoS for VoIP and video streaming over LTE networks.

In [12], we defined time-dependent metrics temporary Received Signal Strength Indicator (RSSI), the instantaneous value of Packet Loss Rate (PLR), an instantaneous value of throughput—*R*, and per-streams metrics the mean values of PLR, throughput *R*, and traffic percentage for neighbor discovery *Rm_hello*, and for sensing *Rm_sensing*. Some MOEs were already presented by us, in [10] a command capability factor (C2F) was proposed to evaluate the network performance for an operational purpose. C2F is a normalized coefficient proportional to the difference between available bandwidth among investigated nodes and required bandwidth for command services.

The instantaneous value of stream throughput (*R*(*t*)) of the *r*-th stream between *i*-th and *j*-th node [bits/s] should be calculated based on the number of bits received by the destination node (per stream) from the source node in predefined time intervals:

(1) $$R_{ij}^{r}\left(t\right)\;=\frac{Nb_{ij}^{r}\left(t\right)}{\Delta t},$$

where: $Nb_{ij}^{r}\left(t\right)$—number of bits correctly sent in Δt intervals [sec], in *r*-th stream between *i*-th and *j*-th node registered at the end of each interval [bits], $\Delta t_{}$ jumping window size for accepted/not accepted sessions statistic.

If $Rtm_{ij}^{r}$ represents the mean throughput of the *r*-th stream between *i*-th and *j*-th node, then the global value of throughput (*Rg*) in the network [bits/s] can be calculated as follows:

(2) $$Rg=\frac{\sum_{i=1}^{N}\sum_{j=1}^{N}\sum_{r=1}^{n}Rtm_{ij}^{r}}{m},$$

where: $Rtm_{ij}^{r}$—mean throughput of the *r*-th stream between *i*-th and *j*-th node [bits/sec], *n*—number of streams between *i*-th and *j*-the node [-], *m*—global number of streams during network operation [-], *N*—number of nodes in the network [-].

In this report, the presented results refer to three types of services, that is, PTT (multicast voice transfer), UDP and BFT (data transfer) services. Based on ITU-T and IETF recommendations [50], the quality measures for the aforementioned services are Packet Loss Rate, Packed Delay Rate (*PDL*), and Jitter (*J*) that is a variation of end-to-end packet delay. A percentage of session success as MOE metrics are used to compare the source to destination performance for the three above-mentioned traffic cases [12].

Instantaneous values of packet loss rate $PLR_{ij}^{r}\left(t\right)$ of the *r*-th stream between *i*-th and *j*-th node, is calculated based on the statistics collected in the destination node (number of received packets), and source node (number of sent packets), using the following formula:

(3) $$PLR_{ij}^{r}\left(t\right)\;=\frac{L_{ij}^{r}\left(t\right)}{D_{ij}^{r}\left(t\right)}\;,$$

where $L_{ij}^{r}\left(t\right)$—number of lost packets in Δt intervals registered at the end of each interval, during *r*-th stream transmission between *i*-th and *j*-th node, $D_{ij}^{r}\left(t\right)$—the number of packets generated by *i*-th node and sent to *j*-th node in Δt.

Based on these values, a global statistic representing the mean value of PLR for all IP streams in the network is calculated after simulation, where PLR of the stream means the percentage of IP datagrams received by the destination node in relation to the number of packets sent by the source node. For each source-destination node, the mean value of PLR per source-destination stream (PLRm) can be calculated. All values of PLRm should be collected in the DB. The global value PLRg is then calculated as

(4) $$PLRg=\frac{\sum_{i=1}^{N}\sum_{j=1}^{N}\sum_{r=1}^{n}PLRm_{ij}^{r}}{m},$$

where: $PLRm_{ij}^{r}$—mean value of packet loss rate in *r*-th stream between *i*-th and *j*-th node, n—number of streams between *i*-th and *j*-the node, m—global number of streams during network operation, N—number of nodes in the network.

The IP packet end-to-end delay metric is used to verify the behavior of the network when transporting this type of traffic. To calculate this metric, the difference between the time a packet arrives at its destination and the time it was transmitted must be calculated. This metric is collected separately for each source, destination pair and traffic type. Both unicast and multicast/broadcast packets generated are considered for this metric.

The IP packet end-to-end delay variation metric computes the variation of the IP packet end-to-end delay metric and is measured in seconds. It is post-processed, using the collected values of the IP packet end-to-end delay metric to calculate the standard deviation.

For voice transmissions (VoIP and PTT), the following decision function is used:

(5) $$Good_{ijk}=\left\{\begin{array}{l} 1\;if\;PLR_{ijk}\left(t\right)\;<PLR_{max}\|\;\cup \;J\;\left(t\right)\;<J\_max\;\cup PDL_{ijk}\left(t\right)\;<PDL\_max\;\\ \;\;0\;otherwise \end{array}\right.\;,$$

where $PLR_{ijk}\left(t\right)$, $J_{ijk}\;\left(t\right),$ *PDL_ijk_* is mean value of jitter and packet delay of *k* packets in *ij* transmission, and max means limit, unacceptable values.

Percentage of success (accepted) sessions is computed as the ratio of well-received to all transmitted packets:

(6) $$Pa=\frac{\sum_{i=1}^{N}\sum_{j=1}^{N}\sum_{k=1}^{n}Good_{ijk}^{}}{\sum_{i=1}^{N}\sum_{j=1}^{N}\sum_{k=1}^{n}All_{ijk}^{}},$$

where Pa is defined separately for two classes of service RT and NRT.

The percentage of PTT multicast sessions finished with success for a given node. It is based on the $M_{SF}$ metric presented in the previous subsection.

(7) $$Pa=\frac{\sum M_{SF}\;\mathrm{for}\;\mathrm{the}\;\mathrm{node}\;\mathrm{of}\;\mathrm{interest}}{\mathrm{Number}\;\mathrm{of}\;\mathrm{PTT}\;\mathrm{multicast}\;\mathrm{sessions}\;\mathrm{of}\;\mathrm{the}\;\mathrm{node}\;\mathrm{of}\;\mathrm{interest}},$$

where $M_{SF}$ is multicast session success which is 1 if multicast sessions proportion is $\geq N_{L,min}$.

For NRT the rate of correctly received messages to all sent messages for each session is compared with the predefined minimum value for example, 0.95.

### 6.2. Simulation Results

**Figure 16:**
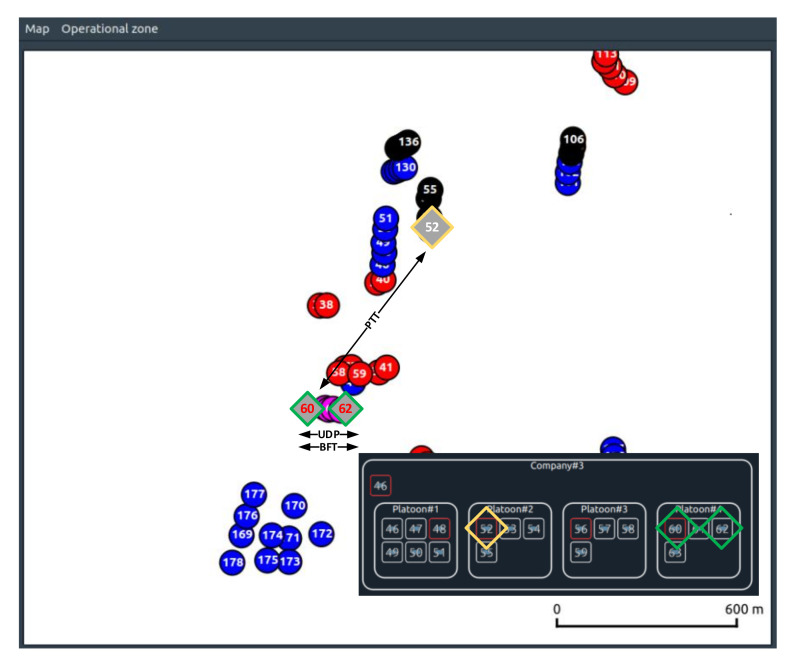
Command relation between 60–62 nodes.

Below, the results obtained will provide a detailed analysis of selected links as examples between nodes 60 and 62 belonging to the support platoon (Platoon#4) of the 1st mechanized company (Company#3) as is shown in Figure 16.

The following sessions were considered: a PTT session set up between 25 and 30 s of simulation, a UDP session running between 20 and 50 s of simulation, and a BFT session running between 20 and 60 s. The volume of traffic generated is consistent with the assumptions presented in the previous section of this paper and the UDP and BFT sessions compete for access to shared resources.

**Figure 17:**
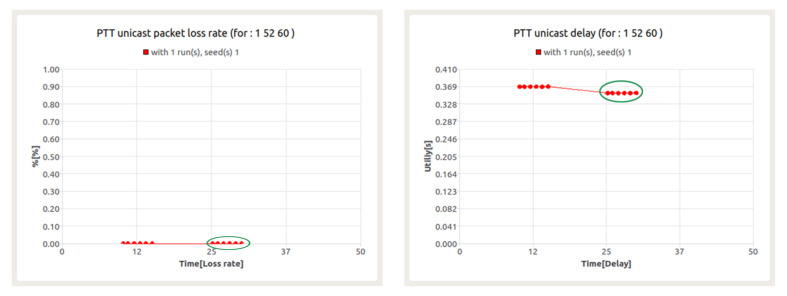
Packet loss ratio (**left**) and e2e delay (**right**) for PTT session—node 60.

The results shown in Figure 17 for the PTT service indicate its correct implementation in a complex simulation environment. The value of packet loss is at the level of 0% and the average delay remains at the value equal to about 350 ms (marked in green circle). The obtained values are within the set of acceptable values defined for the PTT service. It should also be noted that the PTT service is treated as a background service to evaluate the behavior of other services.

**Figure 18:**
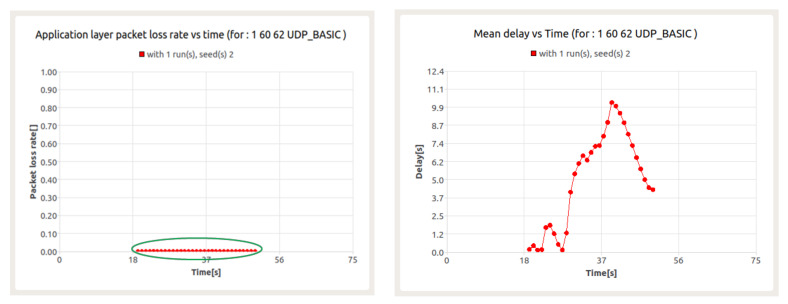
Packet loss ratio (**left**) and e2e delay (**right**) for UDP session in the presence of background traffic—node 60.

The results presented in the next figure (Figure 18) are for UDP service execution in the presence of background traffic. As can be seen, the packet loss ratio is equal to 0% for the entire duration of the execution of the service. In turn, the delay value oscillates between 0.1–10.1 s. Taking into account the quality criteria defined for that service it should be concluded that it is realized without any disturbances. However, the latency value recorded during the simulation shows its sharp increase from 28th second, which continues, until about 40th second.

Let’s try to analyze the obtained values of delay. Taking into account the fact that the transmission is realized with the use of UHF waveform (so-called Basic UHF Waveform), one can assume TDMA frame duration equal to 75.197 ms. Thus, the average delay between when a packet is first generated at the application layer and when a MAC layer message is sent is equal to 190 ms. This duration must still be supplemented by the duration of the MAC frame, so the total delay will be 300 ms in this case.

Note that the node under consideration belongs to the Comp_BMS network and supports intra- and inter-cluster flows. It is also a gateway node, which makes the initial delay double, and thus takes the value of about 1.2 s. However, the calculated delay value applies only to the first packets of a traffic flow. Unfortunately, for subsequent packets, the calculations performed can no longer be taken into account due to the possibility of congestion. The congestions occur in two different situations. In the DATA part of the TDMA frame, when too much traffic has to be transmitted to the Communication Node, or in the RAS (Random Access Slot) portion of a TDMA frame due to collisions, that is, when different CNs select the same RAS slot at the same time. These collisions take place when the same slot is assigned to different nodes which can happen if the nodes are initially far from each other and they are approaching. In both cases, no packet can be transmitted and the delay increases.

In the case under consideration, the reason for the congestion and, at the same time, the increase in latency is the amount of data needed to be sent by node 60, which handles the data transfer to node 62 within the intra-cluster session and, at the same time, is the gateway for nodes 61 and 63.

**Figure 19:**
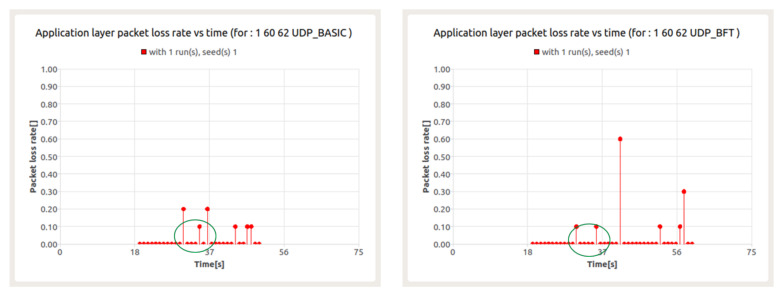
Packet loss ratio for UDP (**left**) and BFT (**right**) sessions in the presence of background traffic—node 60.

**Figure 20:**
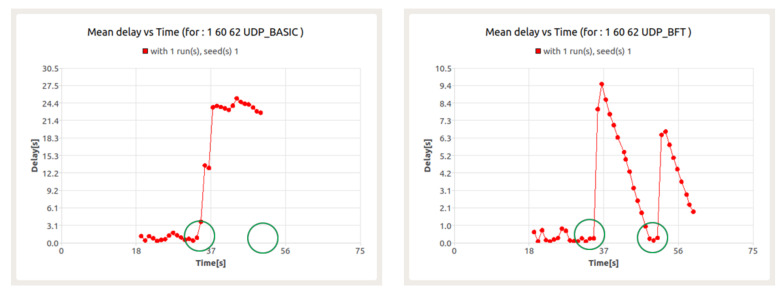
Delay for UDP (**left**) and BFT (**right**) sessions in the presence of background traffic—node 60.

The above conclusion is confirmed by the next series of results presented. Figure 19 shows the loss ratio for UDP service in the presence of BFT traffic for node 60. In turn, Figure 20 refers to the packet transfer delay for the mentioned services.

The results presented in Figure 20 show the occurrence of packet loss at the 31st second and between the 34th and 36th seconds. The resulting losses are a consequence of collisions in data transfer between UDP session and BFT session. For both sessions, this leads to a significant increase in latency.

**Figure 21:**
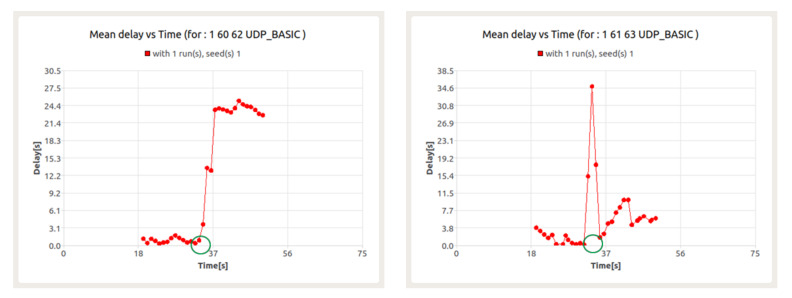
Delay for UDP session in the presence of background traffic between nodes 60–62 (**left**) and between nodes 61–63 (**right**) with node 60 as a gateway.

However, while the latency in the BFT session quickly returns to acceptable levels, it persists for the UDP session until the end of the session. This is due to the previously mentioned role of node 60 as a gateway for CNs 61 and 63 (Figure 21).

A PTT session is considered a success if: (1) its loss rate is lower than 3%, (2) its average delay is lower than 500 ms, and (3) its average jitter is lower than 50 ms. Above values are slightly higher than those recommended in [51] for wired IP connections, because of expected jamming and limited spectral resources for military purposes. For other services the accepted parameters are as follows:

- loss ratio lower then 5%
- delay lower then 20 s
- jitter has no impact.

**Figure 22:**
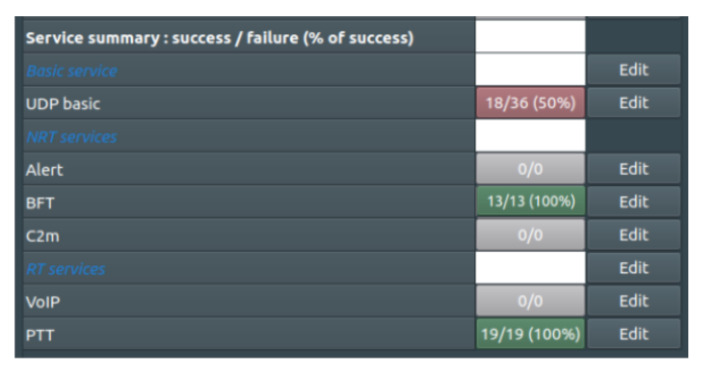
Summary of services successful realization.

The last line of Figure 22 indicates that all VHF PTT sessions (100%) are successful. The figure shows a summary of the results of the simulation during which, according to the assumed scenario, subsequent services using the VHF and UHF waveforms were launched. It is worth noting that in addition to the PTT sessions, the BFT sessions were also implemented in the set. The significant degradation of UDP services is due to resource sharing with BFT services.

**Figure 23:**
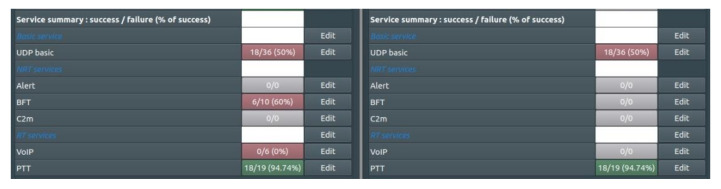
Summary of services realization in the case without an integrated solution.

For comparison, the results of a simulation in which the integrated solution was not used are shown (Figure 23). In two consecutive experiments, significant degradation in the performance of the UDP service was obtained, which is precisely the result of the lack of the integrated solution shown in this paper.

## 7. Conclusions

The High-Fidelity simulation of the mobile ad hoc network is a critical step of real system design, enabling verification of assumptions, modeling of real radio environment, used devices, protocol stacks, and proposed mechanisms for spectrum management. Because spectrum usage is closely related to the network structure, number of nodes and their layout, the available spectral resources are the main factor limiting the use of particular services, and the network performance under variable traffic should be investigated.

Because MANET network parameters depend on properties of particular radio links, the aggregated traffic models are not relevant for this application. Instead, authors propose to use the source traffic models, including both real-time and non-real-time services using different transport protocols.

Activity of specific nodes is described by their users’ profiles, with predefined statistical parameters, coming from real exercises.

Effective modeling of complex military communication systems requires:

- Nodes’ traffic generation for multiservice profiles in complex and dynamic battlefield operation.
- Combination of traffic generated inside particular networks taking into account multi-address traffic for messages senders and recipients.
- Combination of traffic in heterogeneous networks with defined users groups if the node can be a member of various groups.

The elaborated model is devoted to these issues and is developed for multinetwork communication systems.

The traffic generator concept was verified using Matlab simulations for simple scenario, and next it was defined for a High-Fidelity MANET simulator. The achieved results show that for complex operational scenarios the performance of the mobile network increases thanks to effective use of both central and distributed DSM.

Simulation results, presented in Section 6, show that the implemented services effectively use the available spectrum resources of the system in accordance with the assigned traffic classes. With respect to the PTT service, this means maintaining an acceptable end-to-end delay of 350 msec and no packet loss. On the other hand, in the case of the BFT service, which shares resources with the UDP service, competition for access to resources is observed. This causes a temporary increase of delays (6–9 s) and packet losses for the BFT. However, the indicated fluctuations, thanks to the applied solutions, disappear in a short period of time that further enables providing of services with acceptable quality. It should also be noted that the obtained results depends on tasks performed by particular CN nodes, acting as gateways for neighboring CNs (for example node 60 in the simulated scenario). Finally, it can be stated that the use of DSM and cognitive solutions leads to significant increase of the realized services quality, and spectral efficiency of MANET.

As presented solution is unique, our research results cannot be compared with other published solutions devoted to the civilian purposes. The proposed simulation tool has been verified and validated through a series of tests and experiments. It is flexible and can handle different types of crisis management and public security scenarios.

In our next studies, we plan to compare results that will be obtained where more advanced propagation models will be used. The further works are also aimed to fully integrate the proposed traffic generator with MAENA simulator and to enable the adaptation of users’ profiles parameters according to dynamically changing policies according to different phases of operation. Moreover, implementation of predefined communication procedures, being sequences of multiple exchanges of information are planned.

## Figures and Tables

**Table 1:** Military MANET services characteristics.

Name of Service	Type of Service	Type of Transfer	Type of Transm.	Size [kB]/Required Bit Rate [kbps]	Protocols
Voice	RT	CO	Packets streams	2.4/16 kbps	UDP
Video	RT	CO/CL	Bit-stream		UDP, UDP/RTP
- Low - Medium - High				17–33 kB 200–500 kB 400–1000 kB	
BFT	NRT	CL	Data blocks	0.2–2 kB	UDP
Alert	NRT	CL	Data blocks	0.2–2 kB	
Chat	NRT	CL	Data blocks		TCP
Formatted messages Free text Short messages				10 kB 2 kB 160 B	
Email	NRT	CL	Data blocks		TCP
Formatted messages Formatted messages with attachments				5–50 kB 5–50 + 500–2000 kB	
C2 maps:	RT	CL	Data blocks		UDP
- tactical overlay - thematic maps Pictures: Sensor data:				500 kB 2000 kB 17–33 kB to 0.2 kb/s	

**Table 2:** Simulation parameters.

Parameter	Description
No. of nodes	183
No. of UHF networks	4
No. of VHF networks	28
No. of radio interfaces for each node	from 2 to 4
No. of UHF interfaces	183
No. of VHF interfaces	236
No. of UHF frequencies	40
No. of VHF frequencies	1500
No. of PTT sessions	19 sessions
No. of BFT sessions	13 sessions
No. of UDP sessions	36 sessions
PTT traffic	each session takes 5 s, repetition period 10 s.
BFT traffic	20 kb/s rate, each session takes 40 s.
UDP traffic	500 b/s, 40 kb/s, 160 kb/s—each session takes about 30 s.
Path loss model	exponential path losses (𝛼 = 3.5)
Size of Initial Hopset	VHF: 20 frequencies UHF: 4 frequencies
IP routing	BEUN (Baseline Extended Upper NET)—routing protocol developed for MANETs
Area of operation	8.2 km × 6.0 km
Order of Battle	1 mechanized battalion, 4 companies, 18 platoons
Simulation time	100 s.

## Data Availability

Not applicable.

## Abbreviations

The following abbreviations are used in this manuscript:

AI	Artificial Intelligence
BFT	Blue Force Tracking
BMS	Battlefield Management System
BW	Basic Waveform
C2	Command and Control
C4ISR	Command, Control, Communications, Computers, Intelligence, Surveillance and Reconnaissance
CBR	Constant Bit Rate
CH	Cluster Head
CL	Connection-less
CMMPP	Coupled Markov Modulated Poisson Process
CN	Communication Node
CO	Connection-oriented
COP	Common Operational Picture
CORASMA	Cognitive radio for dynamic spectrum management
CR	Cognitive Radio
CVSD	Continuously Variable Slope Delta
DSA	Dynamic Spectrum Access
DTM	Digital Map of Terrain
DTEM	Digital Terrain Elevation Model
DSA	Dynamic Spectrum Management
DSM	Dynamic Spectrum Management
EDA	European Defense Agency
FER	Frame Error Rate
FH	Frequency Hopping
GIG	Global Information Grid
GW	Gateway
HDR	High Data Rate
HMI	Human Machine Interface
HMM	Hidden Markov Model
IED	Improvised Explosive Device
iMANET	Internet-based MANET
inVANET	Intelligent VANET
IoT	Internet of Things
IRC	Inter-Roadside Communication
IVC	Inter-Vehicles Communication
LOP	Local Operational Picture
M2M	Machine to Machine
MAC	Medium Access Control
MAENA	Multi bAnd Efficient Networks for Ad hoc communications
MANET	Mobile ad hoc networks
MANET-CR	Mobile ad hoc networks with Cognitive Radio
ML	Machine Learning
MMPP	Markov Modulated Poisson Process
MOE	Metrics of Effectiveness
MOP	Metrics of Performance
NCN	Non-Cooperative Nodes
NCW	Network-Centric Warfare
ON	Ordinary Nodes
ORBAT	Order of Battle
OSA	Opportunistic Spectrum Access
PDL	Packet Delay
PER	Packet Error Rate
PLC	Power Line Communications
PLR	Packet Loss Rate
PTT	Push-to-Talk
QoS	Quality of Service
RECON	Recognition
RN	Regular Node
RTP	Real-time Transport Protocol
RSSI	Received Signal Strength Indicator
SA	Situational Awareness
TCP	Transmission Control Protocol
UAV	Unmanned Aerial Vehicle
UDP	User Datagram Protocol
UGS	Unmanned Ground Sensor
UHF	Ultra High Frequency
VANET	Vehicular Ad Hoc Network
VBR	Variable Bit Rate
VHF	Very High Frequency
VioIP	Video over IP
VoIP	Voice over IP
VRC	Vehicle to Roadside Communication
WB	Wide Band
WSN	Wireless Sensors Networks

## References

[1] Gajewski P., Lopatka J., Lubkowski P. Traffic Models for Military MANET Simulation. Proceedings of the 2021 Signal Processing Symposium (SPSympo).

[2] Brubank J.L., Chomento P.F., Haberman B.K., Kasch W.T. (2006). Key Challenges of Military Tactical Networking and the Elusive Promise of MANET Technology. IEEE Commun. Mag..

[3] Kaur S., Beri R., Singh S. (2016). A Survey on Ad Hoc Network (MANET). Int. J. Sci. Res. Dev..

[4] Haykin S. (2005). Cognitive Radio: Brain-Empowered Wireless Communications. IEEE J. Sel. Areas Commun..

[5] Song M., Xin C., Zhao Y., Cheng X. (2012). Dynamic spectrum access: From cognitive radio to network radio. IEEE Wirel. Commun..

[6] Ghosh G., Das P., Chatterjee S. (2014). Cognitive Radio And Dynamic Spectrum Access–A Study. Int. J. Next-Gener. Netw..

[7] Popoola J.J., van Olst R. (2014). A Survey on Dynamic Spectrum Access via Cognitive Radio: Taxonomy, Requirements, and Benefits. Univers. J. Commun. Netw..

[8] Lopatka J., Dołowski J., Grochowina B., Bryś R. Efficiency Assessment of Coordinated Hierarchical Dynamic Spectrum Management for Mobile AD HOC Networks. Proceedings of the 38th International Business Information Management Association Conference (IBIMA).

[9] Rose L., Massin R., Vijayandran L., Debbah M., LeMartret C.J. (2014). CORASMA Program on Cognitive Radio for Tactical Networks: High Fidelity Simulator and First Results on Dynamic Frequency Allocation. Proceedings of the MILCOM 2013—2013 IEEE Military Communications Conference.

[10] Szmit G., Dołowski J., Łopatka J. Distributed channel selection for hierarchical cognitive radio networks. Proceedings of the MILCOM 2015—2015 IEEE Military Communications Conference.

[11] Gajewski P., Lopatka J. MANET Cognitive Radio realtime testbed for Dynamic Spectrum Access. Proceedings of the AFRICON—2017.

[12] Lopatka J., Gajewski P., Malon K., Krygier J. Performance Monitoring of Cognitive Radio Mobile ad hoc Network with Dynamic Spectrum Access. Proceedings of the AFRICON—2017.

[13] Kaszuba-Chęcińska A., Chęciński R., Gajewski P., Łopatka J. (2021). Cognitive Radio MANET Waveform Design and Evaluation. Sensors.

[14] Tatugate A.A., Jadhav S.R. Tactical Communication System using MANET and WSN. Proceedings of the International Conference on Advances in Engineering and Technology—2014 (ICAET—2014).

[15] Naderi E., Asrari A. Hardware-in-the-Loop Experimental Validation for a Lab-Scale Microgrid Targeted by Cyberattacks. Proceedings of the 2021 9th International Conference on Smart Grid (icSmartGrid).

[16] Adoni K.A., Tavildar A.S., Warhade K.K. (2020). Random Black Hole Attack Modelling and Mitigation Using Trust-Confidence Aware OLSR in MANETs for Private Data Communications. Int. J. Sens. Wirel. Commun. Control.

[17] Srilakshmi U., Veeraiah N., Alotaibi Y., Alghamdi S., Khalaf O.I., Subbayamma B.V. (2021). An Improved Hybrid Secure Multipath Routing Protocol for MANET. IEEE Access.

[18] Bondada P., Samanta D., Kaur M., Lee H.-N. (2022). Data SecurityBased Routing in MANETs Using Key Management Mechanism. Appl. Sci..

[19] Yamini K.A.P., Stephy J., Suthendran K., Ravi V. (2022). Improving routing disruption attack detection in MANETs using efficient trust establishment. Trans. Emerg. Telecommun. Technol..

[20] Saleh S.A., Zuhairi M.F., Dao H. (2020). A Comparative Performance Analysis of Manet Routing Protocols in Various Propagation Loss Models Using NS3 Simulator. J. Commun..

[21] Rath M., Pattanayak B.K. (2021). Performance evaluation of optimised protocol in MANET. Int. J. Inf. Comput. Secur..

[22] Ben Brahim G., Mohammad N., El-Hajj W., Parr G., Scotney B. (2022). Performance evaluation and comparison study of adaptive MANET service location and discovery protocols for highly dynamic environments. EURASIP J. Wirel. Commun. Netw..

[23] Abdan M., Seno S.A.H. (2022). Machine Learning Methods for Intrusive Detection of Wormhole Attack in Mobile Ad Hoc Network (MANET). Wirel. Commun. Mob. Comput..

[24] Erciyes K., Dagdeviren O., Cokuslu D., Yılmaz O., Gumus H. (2012). Modeling and Simulation of Mobile Ad hoc Networks. Mobile Ad Hoc Networks.

[25] Martinez F.J., Toh Ch K., Cano J.-C., Calafate C.T., Manzoni P. (2011). A survey and comparative study of simulators for vehicular ad hoc networks (VANETs). Wirel. Commun. Mob. Comput..

[26] Jie L., Wang L. (2019). Simulation tools for MANETs: A systematic survey. Int. J. Comput. Sci. Eng. Surv..

[27] Bednarczyk W., Gajewski P. (2015). Performance of distributed clustering with weighted optimization algorithm for MANET Cognitive Radio. Proceedings of the 2015 International Conference on Military Communications and Information Systems (ICMCIS).

[28] Skokowski P., Malon K., Lopatka J. (2022). Building the Electromagnetic Situation Awareness in MANET Cognitive Radio Networks for Urban Areas. Sensors.

[29] Kaszuba-Chęcińska A. Propagation Loss Prediction at 300 MHz in Suburban Environment. Proceedings of the 37th International Business Information Management Association Conference (IBIMA).

[30] Chandrasekaran B. Survey of Network Traffic Models. https://www.cse.wustl.edu/~jain/cse567-06/traffic_models3.html.

[31] Mohamed A.M., Agamy A.F. (2011). A Survey on the Common Network Traffic Sources Models. Int. J. Comput. Netw..

[32] Garcia A.E., Hackbarth K.D., Brand A., Lehnert R. Analytical Model for Voice over IP Traffic Characterization. https://www.academia.edu/6013060/Analytical_Model_for_Voice_over_IP_traffic_characterization.

[33] Haroun R.F., Takiedeen A.E., Abdelhay E.H., Mohamed A.M. (2018). Performance Evaluation of QoS for VoIP and Video Streaming ove LTE Networks. Int. J. Sci. Eng. Technol..

[34] Choi S.W., Song Y.-S., Shin W.-Y., Kim J. (2019). A Feasibility Study on Mission-Critical Push-to-Talk: Standards and Implementation Perspectives. IEEE Commun. Mag..

[35] Chang L.-H., Sung C.-H., Chu H.-C., Liaw J.-J. (2009). Design and implementation of the push-to-talk service in ad hoc VoIP network. IET Commun..

[36] Alakiri O.H., Oladeji A., Benjamin C.B., Okolie C.C., Okikiola M.F. (2014). The desirability of pareto distribution for modeling modern internet traffic characteristics. Int. J. Nov. Res. Eng. Appl. Sci..

[37] Ferreira J.C.V., Contreras M.F., Sierra J.E. (2018). Modelling and Characterization Traffic Voice, Video, Data and Telemetry under Pareto Distribution-Oriented Networks have on Power Line Communications. Indian J. Sci. Technol..

[38] Naveenrai M., Karthick P.V., Karthic S. (2018). Traffic Analysis Using MANET in Wireless Sensor Network. Int. J. Pure Appl. Math..

[39] Conti M., Li Q.Q., Maragno A., Spolaor R. (2018). The Dark Side(-Channel) of Mobile Devices: A Survey on Network Traffic Analysis. IEEE Commun. Surv. Tutor..

[40] White Paper—”A 5G Traffic Model for Industrial Use Cases”, 5G Alliance for Connected Industries and Automation, November 2019. https://5g-acia.org/wp-content/uploads/2021/04/WP_5G_5G_Traffic_Model_for_Industrial_Use_Cases_22.10.19.pdf.

[41] Haryadi S. (2018). Telecommunication Traffic Unit and Traffic Mathematical Model. Telecommunication Traffic: Technical and Business Consideration.

[42] (2011). 3GPP TR 37.868. Study on RAN Improvements for Machine Type Communications, Releases 11. https://fdocuments.in/document/3gpp-tr-37868-v1100-2011-09.html.

[43] Osahenvemwen O.A., Edeko F.O., Emagbetere J. (2012). Traffic Modeling in Mobile Communication Networks. Int. J. Comp. App..

[44] Laner M., Svoboda P., Nikaein N., Rupp M. (2013). Traffic Models for Machine Type Communications. ISWCS 2013: Proceedings of the Tenth International Symposium on Wireless Communication Systems, Ilmenau, Germany, 27–30 August 2013.

[45] Ji L., Yin X., Shi X., Wang Z. (2008). Conversational Model Based VoIP Traffic Generation. Proceedings of the International Conference on Networking and Services (ICNS ‘07).

[46] Doci A., Barolli L., Xhafa F. (2009). Recent Advances on the Simulation Models for Ad Hoc Networks: Real Traffic and Mobility Models. Scalable Comput. Pract. Exp..

[47] Zeng Y., Chen T.M. (2004). Measurement-based Real-time Traffic Model Classification. Proceedings of the 2004 IEEE International Conference on Communications.

[48] Zhao Y., Mao S., Neel J., Reed J. (2009). Performance Evaluation of Cognitive Radios: Metrics, Utility Functions, and Methodology. Proc. IEEE.

[49] Kumari C.S., Setty P. (2017). Performance Analysis of Cognitive Radio Networks (IEEE 802.22) for Various Network Traffics. Int. J. Recent Innov. Trends Comput. Commun..

[50] ITU-T Recommendation Y.1540: “Internet Protocol Data Communication Service—IP Packet Transfer and Availability Performance Parameters. https://www.google.de/#q=ITU-T+Recommendation+Y.1540.

[51] ITU-T Recommendation Y.1541: “Network Performance Objectives for IP-Based Services”. https://www.itu.int/rec/T-REC-Y.1541/en.

